# Patterns of sedentary behavior in adults: A cross-sectional study

**DOI:** 10.3389/fcvm.2023.1116499

**Published:** 2023-03-13

**Authors:** Gustavo O. Silva, Paolo M. Cunha, Max D. Oliveira, Diego G. D. Christofaro, William R. Tebar, Aline M. Gerage, Hélcio Kanegusuku, Marilia A. Correia, Raphael M. Ritti-Dias

**Affiliations:** ^1^ Postgraduate Program in Rehabilitation Sciences, Universidade Nove de Julho (UNINOVE), Sao Paulo, Brazil; ^2^ Instituto Israelita de Ensino e Pesquisa Albert Einstein (IIEP), Hospital Israelita Albert Einstein, Sao Paulo, Brazil; ^3^ Faculty of Science and Technology, Sao Paulo State University (Unesp), Presidente Prudente, Brazil; ^4^ Center for Clinical and Epidemiological Research, University Hospital, University of Sao Paulo, Sao Paulo, Brazil; ^5^ Physical Education Department, Federal University of Santa Catarina, Florianopolis, Brazil

**Keywords:** physical activity, cardiometabolic health, exercise, accelerometer, sitting

## Abstract

**Introduction:**

Sedentary behavior (SB) has been associated with adverse health outcomes, however, it is not completely clear whether total time in SB during the day or prolonged uninterrupted SB are interrelated. The aim of the current study was to describe the different patterns of SB of adults, their relationships, and associated factors.

**Methods:**

The sample included 184 adults aged ranging from 18 to 59 years old. SB was objectively measured by an accelerometer and the following SB pattern parameters were obtained: total time in sedentary bouts, mean time of sedentary bouts, and total time in sedentary breaks. Demographic data (age and sex), anthropometry [weight, height, body mass index (BMI)], blood pressure (BP), medical history (self-reported comorbid conditions), and cardiac autonomic modulation, were assessed to identify factors associated with SB. Multiple linear regressions were used to analyze the relationship between SB parameters and the associated factors.

**Results:**

The parameters of SB indicated 2.4 (0.9) h/day for total time in sedentary bouts, 36.4 (7.9) min for the mean time of sedentary bouts, and 9.1 (1.9) h/day for the total time in sedentary breaks. Multiple adjusted regression indicated that age was the only factor associated with SB patterns (*p* < 0.05) after adjustment for confounding variables (sex, age, BMI, dyslipidemia, systolic and diastolic BP). Young adults (18–39 years old) spent more time in sedentary bouts and less time in uninterrupted sedentary bouts compared to middle-aged adults (40–59 years old) (2.58 (0.88) h/day vs. 2.13 (0.90) h/day, respectively; *p* = 0.001 and 34.5 (5.8) min 18–39 years old vs. 38.8 (9.6) min 40–59 years old; *p  *≤ 0.001; respectively). The total time in sedentary breaks was similar between age groups (*p* = 0.465). The total time in sedentary bouts was significantly correlated with the mean time of sedentary bouts (*r* = −0.58; *p *≤ 0.001), and with the total time in sedentary breaks (*r* = −0.20; *p* = 0.006). The mean time of sedentary bouts was significantly related to the total time in sedentary breaks (*r* -= 0.19; *p* = 0.007).

**Discussion and Conclusion:**

In conclusion, age seems to be a relevant factor associated with sedentary behavior with young adults spending more time in SB and accumulating this behavior in a higher amount of sedentary bouts compared to middle-aged adults.

## Introduction

Sedentary behavior (SB) is characterized by any behavior while awake in a sitting, reclining, or lying posture with an energy expenditure of ≤1.5 metabolic equivalents (1), and high time spent in SB has been associated with adverse health outcomes and risk of mortality from all-causes (2, 3), even in adults that meet guidelines for physical activity (4).

In the literature, some studies have shown that adults spend on average 6–8 h a day in sedentary behavior, which can eventually lead to elevated levels of blood pressure (5), a worsening in vascular function (6) and impaired cardiac autonomic modulation (7), which are risk factors for cardiovascular disease morbidity and mortality, and the emergence and progression of atherosclerotic lesions (8–10).

The physiology behind the harms of SB has been studied in laboratory-controlled studies employing 3–8 h of uninterrupted sitting. The results indicated that prolonged SB promoted impairments in vascular function and increases in blood pressure (11). However, whether this pattern of SB occurs in real-life situations is unclear. In contrast, most epidemiological data about the consequences of SB on health have considered the overall time spent on SB during the day, but the patterns of sedentary time can also consider the duration of the sedentary bouts, with studies suggesting that prolonged, uninterrupted sedentary time is also detrimental (12–16). However, it is not completely clear whether total time in SB during the day and prolonged uninterrupted SB are interrelated. In this study, we describe the different patterns of SB in adults, their interrelationships, and associated factors.

## Materials and methods

### Participants

This cross-sectional study is an exploratory analysis of previous work (17). The sample was comprised of adults aged 18–59 years old, from the city of Santo Anastácio in the southeast of Brazil, as previously described (17). The sample size of 126 participants was calculated based on previous studies (17, 18) and these factors: (a) the population aged 18 years or over in the city of Santo Anastácio is 16,000; (b) the correlation among sedentary behavior parameters and age of *r* = 0.17; (c) 80% power; and (d) 5% alpha error (19). To take into consideration possible errors in reading accelerometry data, misuse of equipment, along with adjustment for confounding factors, we recruited a total of 220 subjects to ensure a sufficient number of participants.

This study was approved by the Ethical Research Committee from Sao Paulo State University—Unesp, under protocol CAAE: 72191717.9.0000.5402. All participants who agreed to participate signed a Written Informed Consent Form.

### Sedentary behavior parameters

Sedentary behavior parameters were measured by the Actigraph GT3X accelerometer (ActiGraph, LLC, Pensacola, FL, United States). The accelerometer was placed on the right side at the waistline and participants were given instructions on how to care for the device and to wear it for the entire day (waking hours), taking off the device only when sleeping and while doing water activities (i.e., hygiene, swimming). Participants were also given instructions to use the accelerometer after receiving it for a minimum of 10 h a day for the following seven days. The 60-second epoch period was considered for this study since it is closest to the pattern of a long-duration activity (20). Consecutive hours of zero counts and days with less than 10 h of monitoring were not considered for the final analysis (21). At least five completed days were considered acceptable for data analysis, three weekdays and two weekend days (22).

Sedentary behavior was defined as activities lower than 200 counts per minute (cpm). The wear time percentage calculation was performed by the division of minutes per week in each physical activity intensity by the total device wear time. Sedentary bouts were defined as periods of uninterrupted sedentary behavior, while sedentary breaks were defined as the time spent in interruptions of sedentary bouts with physical activities (Time not spent being sedentary). For the analyses, we considered:

 - The total time in sedentary bouts: The time spent in sedentary bouts (<200 cpm for at least 10 min) during the day;
 - Mean time in sedentary bouts: The time spent in sedentary bouts (<200 cpm for at least 10 min) divided by the total number of sedentary bouts.
 - The total time in sedentary breaks: The sum of the time spent in physical activities (≥200 cpm for at least 10 min).

**Figure 1:**
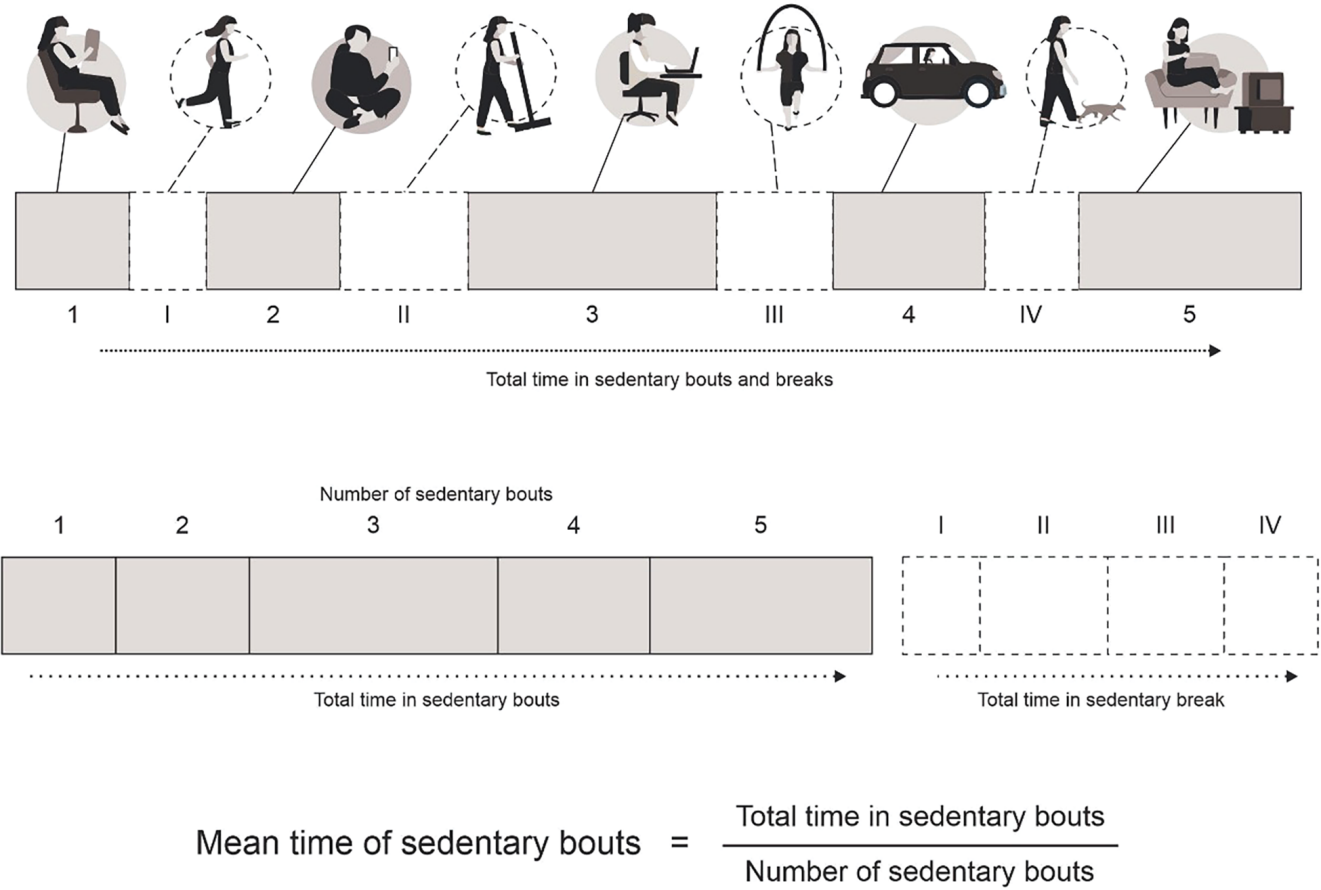
Schematic view of the sedentary behavior variables considered in the analyses.

Figure 1 shows a schematic view of the sedentary behavior variables considered in the analyses.

### Factors associated

Demographic factors (age, sex, diabetes, hypertension, arthritis, obesity, and dyslipidemia) were analyzed to examine their association with SB patterns. The presence of comorbid conditions was assessed by self-report. Weight was measured with a digital scale and height with a stadiometer. Body mass index (BMI) was calculated by the following formula: body weight/height^2^. The participants with BMI values up to 24.99 kg/m^2^ were classified as having normal weight, the ones with values from 25.00 to 29.99 kg/m^2^ as being overweight, and the ones with a BMI ≥ 30 kg/m^2^ as obese. Waist circumference was measured at the midpoint between the iliac crest and the last rib with an inextensible tape with a length of 2 m and an accuracy of 0.1 cm.

Blood pressure was measured with an automatic monitor (HEM-742, Omro Healthcare, Japan). To do so, the participants spent 5 min in the supine position, with the use of an adequate cuff for the arm circumference. Continuous measures were performed with 1 min of the interval among them, on the right arm, until arriving at a difference below 4 mmHg between two measurements. The value used for analysis was the average of the last two measures, as recommended by the American Society of Cardiology (23). This same equipment also provides resting heart rate values together with blood pressure, so the same procedures were used to assess resting heart rate.

Cardiac autonomic modulation assessment was done using the heart rate variability (HRV) analysis. For this assessment, the participants received the following instruction: not to consume beverages containing alcohol or caffeine and not to practice any type of exercise on the previous 12 h before the HRV measurement, in order to prevent any impact on cardiac autonomic modulation during the measurement (24). The HRV was recorded for 30 min, with the subjects resting in the supine position, and maintaining normal breathing during the period of the recording. The HRV indexes were calculated using linear methods and analyzed in the time and frequency domains. The following linear indices were calculated: RMSSD and SDNN (25). The following domains were used for the frequency domain analysis: the low-frequency (LF −0.04 to 0.15 Hz) and high-frequency (HF −0.15 to 0.4 Hz) spectral components in standardized units, representing the relative value of each spectral component in relation to the total power minus the very low-frequency component. All analyses were performed using the Kubios HRV Analysis, version 2.0 (Kupio University, Finland) software, and the Visual Recurrence Analysis, version 4.9 (Eugene Kononov, United States).

### Statistical analysis

Continuous variables were presented as mean and standard deviation, while categorical variables were presented as absolute frequency. Student's *t*-test was used to compare SB (Total time and mean time of sedentary bouts, total time in sedentary breaks) by age group. Multiple linear regression analyses were performed to analyze the relationship between SB, demographic (age, sex, and self-reported comorbid conditions), and cardiometabolic (weight, height, body mass index blood pressure, and cardiac autonomic modulation) factors. Possible confounding variables (Sex, age, BMI, dyslipidemia, systolic and diastolic blood pressure) were tested in the bivariate analyses and all those with a *p*-value <0.20 were entered simultaneously in the final model. Multicollinearity analysis was performed assuming variance inflation factors less than 5, on which in case of not attending to this criteria the variables would be removed from the analysis model. The significance level was set at *p *< 0.05.

## Results

Of the 220 participants recruited, 36 were excluded from the final analysis due to either missing physical activity data or due to significant outlier values due to misuse of the accelerometer device, making a total of 184 participants included in the final analysis. The clinical characteristics, SB, and comorbid conditions of the participants included in the final analysis are described in Table 1. Most participants were women (*N* = 99, 54%) and overweight. Hypertension was observed in 39 participants (21%). The accelerometer was used for 6.7 (1.2) days for 13.9 (1.8) h/day. The parameters of SB indicated 2.4 (0.9) h/day for the total time in sedentary bouts, 36.4 (7.9) min for the mean time of sedentary bouts, and 9.1 (1.9) h/day for the total time in sedentary breaks.

**Table 1:** General sample characteristics (*N* = 184).

Variables	Values
Sex, women (*N*, %)	99, 54
Age (years)	37 (13)
Body mass index (kg/m^2^)	28.4 (5.5)
Systolic blood pressure (mmHg)	122 (16)
Diastolic blood pressure (mmHg)	75 (11)
Heart rate (bpm)	71 (11)
LF/HF	2.4 (2.0)
*Sedentary behavior*
Total time in sedentary bouts (h/day)	2.38 (0.92)
Mean time of sedentary bouts (min)	36.4 (7.9)
Total time in sedentary breaks (h/day)	9.07 (1.93)
Average days of equipment use	6.7 (1.2)
Average time of equipment use (h/day)	13.99 (1.81)
*Comorbidities (N, %)*
Obesity	51, 35
Hypertension	39, 21
Diabetes	7, 4
Dyslipidemia	26, 14
Arthritis	15, 8

Values presented in mean (standard deviation) or absolute and relative frequency (*N*, %). LF/HF, low-frequency/high-frequency ratio.

**Figure 2:**
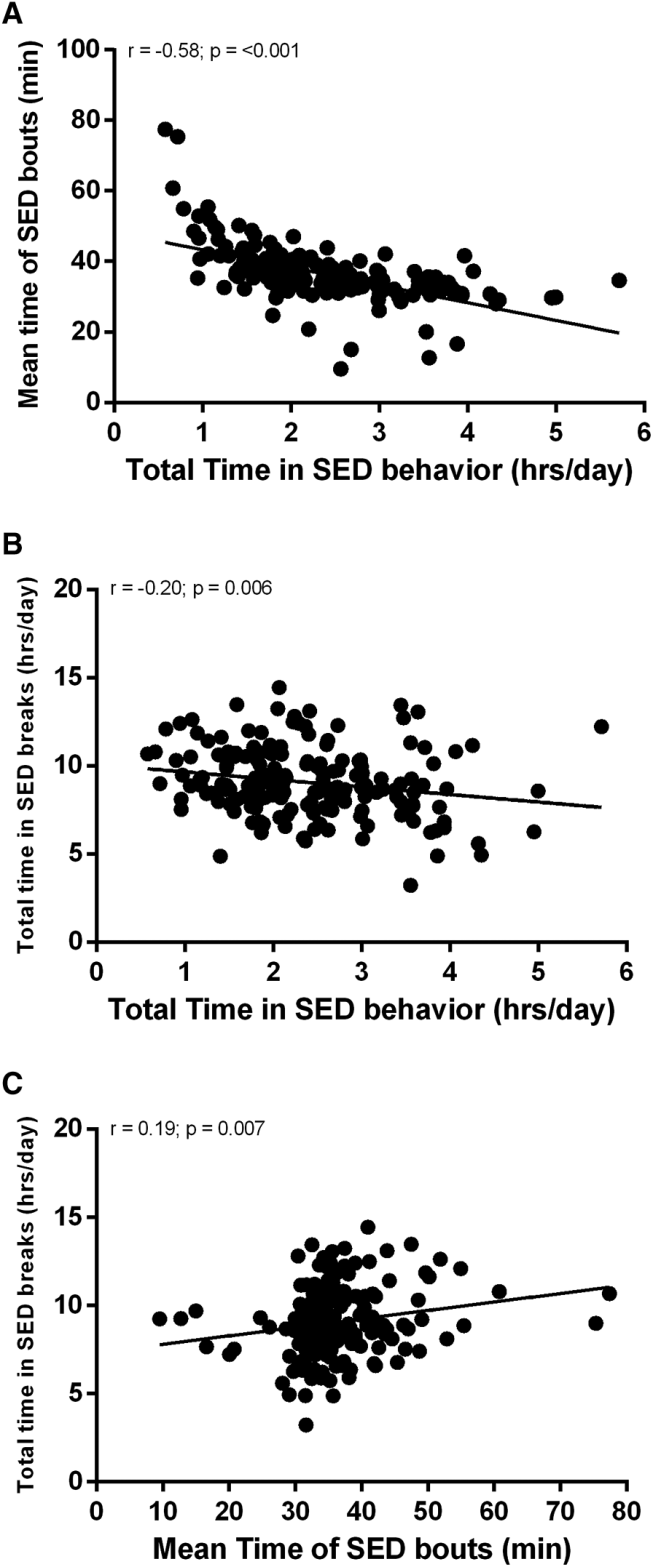
Correlation between different parameters of sedentary behavior.

Figure 2 shows the correlation between different parameters of SB. The total time in sedentary bouts was significantly correlated with the mean time of sedentary bouts (Figure 2A, *r* = −0.58; *p *≤ 0.001) and with the total time in sedentary breaks (Figure 2B, *r* = −0.20; *p* = 0.006). The mean time of sedentary bouts presented a significant correlation with the total time in sedentary breaks (Figure 2C, *r* = 0.19; *p* = 0.007).

Table 2 shows multiple crude and adjusted linear regression analyses of the factors associated with the SB parameters. In the adjusted models, age was the only factor associated with total time in sedentary bouts (*b* = −14.9; SE = 5.9; CI 95%: −26.7, −3.3; *p *= 0.012), mean time in sedentary bouts (*b* = 0.15; SE = 0.06; CI 95%: 0.03, 0.27; *p *= 0.015) and total time in sedentary breaks (*b* = 16.7; SE = 8.4; CI 95%: 0.1, 33.3; *p *= 0.049).

**Table 2:** Crude and adjusted regression of the total time in sedentary bouts, the mean time of sedentary bouts, and the total time in sedentary breaks.

Variables		Crude			Adjusted*		
		*b* (SE)	CI 95%	*p*	*b* (SE)	CI 95%	*p*
Total time in SED bouts	Sex	**−253.8 (126.3)**	**−503.1; −4.6**	**0.046**	−176.9 (136.1)	−446.2; 92.4	0.196
	Age	**−13.7 (4.9)**	**−23.5; −4.1**	**0.006**	**−14.9 (5.9)**	**−26.7; −3.3**	**0.012**
	Dyslipidemia	**−370.9 (179.2)**	**−724.6; −17.3**	**0.040**	−155.7 (183.4)	−518.6; 207.1	0.397
	SBP	−6.9 (4.1)	−15.1; 1.3	0.097	−8.8 (6.6)	−22.0; 4.3	0.187
	DBP	−10.2 (6.3)	−22.8; 2.3	0.110	13.5 (10.1)	−6.7; 33.7	0.186
Mean time of SED bouts	Sex	1.1 (1.2)	−1.3; 3.3	0.391	0.8 (1.4)	−2.0; 3.5	0.593
	Age	**0.14 (0.04)**	**0.06; 0.23**	**0.002**	**0.15 (0.06)**	**0.03; 0.27**	**0.015**
	Dyslipidemia	**3.7 (1.7)**	**0.4; 7.0**	**0.027**	1.0 (1.9)	−2.7; 4.8	0.589
	SBP	0.03 (0.04)	−0.05; 0.11	0.371	0.04 (0.07)	−0.09; 0.18	0.566
	DBP	0.07 (0.06)	−0.05; 0.19	0.247	−0.06 (0.10)	−0.27; 0.15	0.546
Total time in SED breaks	Sex	173.7 (262.9)	−344.6; 692.1	0.509	184.8 (193.6)	−198.2; 567.8	0.342
	Age	9.2 (6.7)	−4.2; 22.6	0.177	**16.7 (8.4)**	**0.1; 33.3**	**0.049**
	Dyslipidemia	75.5 (369.1)	−652.3; 803.2	0.838	−81.9 (260.9)	−597.8; 434.1	0.754
	SBP	−8.5 (9.2)	−26.7; 9.7	0.359	−1.8 (9.5)	−20.5; 16.9	0.850
	DBP	−15.9 (13.9)	−43.6; 11.6	0.255	−7.8 (14.5)	−36.4; 20.8	0.591

*b* (SE), regression coefficient (standard error); CI 95%, confidence intervals 95%; BMI, body mass index; SBP, systolic blood pressure; DBP, diastolic blood pressure.

* Adjusted by sex, age, BMI, dyslipidemia, and systolic and diastolic blood pressure. Reference categories for sex and dyslipidemia were male and presence, respectively.

Bold values mean significant results (*p* < 0.05).

**Figure 3:**
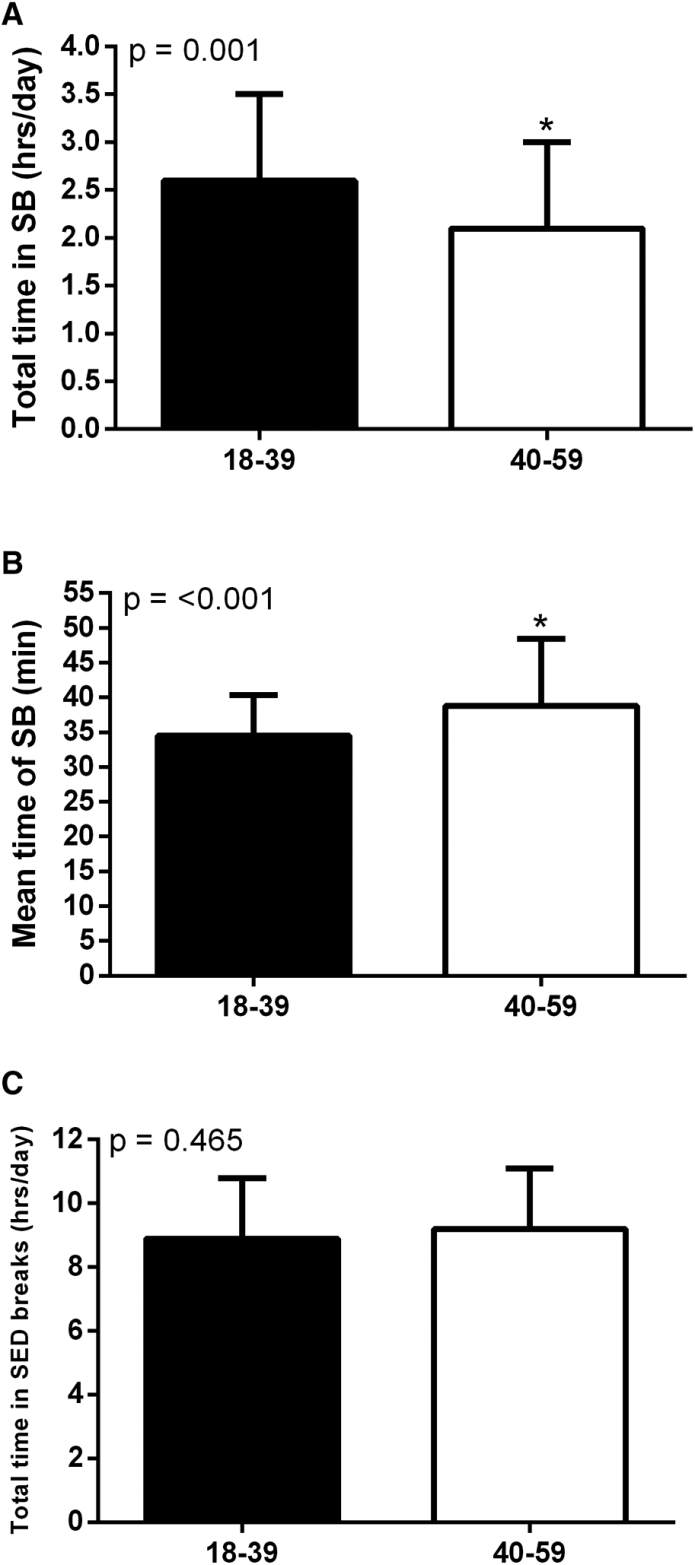
Total time in sedentary bouts (**A**), mean time of sedentary bouts (**B**) and total time in sedentary breaks (**C**) by age group. *Significant difference between age groups.

Figure 3 presents the total time in sedentary bouts (A), mean time of sedentary bouts (B), and total time in sedentary breaks (C) by age groups. Total time in sedentary bouts was higher in young adults (18–39 years old) [2.58 (0.88) h/day] compared to middle-aged adults (40–59 years old) [2.13 (0.90) h/day] (*p* = 0.001). The mean time of sedentary bouts was lower in young compared to middle-aged adults [Young: 34.5 (5.8) min vs. Middle-age: 38.8 (9.6)] (*p* ≤ 0.001). Total time in sedentary breaks was similar between age groups (*p* = 0.465).

## Discussion

The main results of this study were: (i) Adults spend on average 36.4 (7.9) min in each sedentary bout, totaling 2.4 (0.9) h/day in these bouts, and 9.1 (1.9) h/day in sedentary breaks; (ii) the total time sedentary bouts was significantly correlated with the mean time of sedentary bouts and with the total time in sedentary breaks, while the mean time of sedentary bouts was significantly correlated with the total time in sedentary breaks; (iii) age was the only factor associated with SB patterns after adjustment for confounding variables; (iv) young adults are more sedentary than middle-aged adults and spend this behavior in shorter, more frequent sedentary bouts.

The average time that adults spend in sedentary bouts found in the results of the current study is in agreement with a previous study in Americans that found the mean time of sedentary bouts of 30 min (26). These values are higher than those performed in studies analyzing the consequences of sedentary behavior in health, which ranges from approximately 11–25 min per bouts/day (2, 18). These observed discrepancies between studies could have been due to different populations studied, methodology used (such as accelerometer type and data reduction), locations, and differences in sample sizes. These findings reveal the importance of placing SB as an aim for interventions in behavior and also identify populations’ risks and associated factors.

The total time in sedentary bouts was associated with the mean time of sedentary bouts and the total time in sedentary breaks, while the mean time of sedentary bouts was associated with the total time in sedentary breaks. However, even though the SB parameters were associated among themselves, a stronger association was found only between the total time sedentary behavior and the mean time of sedentary bouts, while the other SB parameters did not show such strong associations. This demonstrates that these SB parameters provide different information about SB, and thus measuring one does not provide good enough information about the other. In other words, these SB parameters seem to reflect different information about SB, thus making it important to investigate several different parameters of SB (26).

Age was the only factor associated with SB patterns after adjustment for confounding variables. We found that middle-aged adults spent less time in SB, but accumulate this behavior in longer sedentary bouts compared to young adults. These results show that young adults are more sedentary than their peers and that their sedentary behavior is accumulated through shorter but more frequent sedentary bouts. In this context, it is known that technological advancements have led to an increasingly sedentary lifestyle (27), which could be even more evident in young adults that are more prone to using technology and thus could explain the higher sedentary behavior time found in this age group, accumulated in shorter but more frequent sedentary bouts. These results demonstrate that when accounting for only the time spent in uninterrupted sedentary bouts instead of the overall time spent in sedentary behavior, only age seems to predict these more specific SB patterns and not other factors.

Interestingly, anthropometric and cardiometabolic parameters were not associated with SB patterns. This contrasts with studies indicating a detrimental association between sedentary time and cardiometabolic biomarkers (2, 28–32). For example, a study reported a significant association between total sedentary time and insulin, waist circumference, HDL-cholesterol, C-reactive protein and triglycerides (2). However, these associations accounted for the total time in sedentary behavior, and did not consider the time in uninterrupted, sedentary bouts, which was the case in our study and probably explains the divergencies found. Additionally, Bellettiere et al. found a linear dose-response relationship of sedentary time with cardiovascular disease events and showed that an additional hour of sedentary time was associated with a 12% increase in multivariable-adjusted risk for cardiovascular disease. Moreover, they also showed that women with higher sedentary time and bout durations presented the greatest cardiovascular disease risk (33). This highlights the need for more robust studies investigating the association of objectively-measured SB patterns (i.e., total and mean time of uninterrupted sedentary bouts, sedentary breaks) with anthropometric and cardiometabolic parameters, in order to clarify the true influence of SB patterns on cardiometabolic health, especially since there is robust evidence indicating that breaking up uninterrupted sedentary bouts can lead to many health benefits (33–37).

This study has limitations that can be highlighted. This is a cross-sectional study that precludes cause-effect inference. In addition, we were unable to verify the relationship between SB and other important blood biomarkers (i.e., C-reactive protein, lipoproteins, and others). The sample size included adults from a single small inner city, which can affect the SB patterns. Lastly, we only studied adults and the results cannot be generalized for the elderly population.

In conclusion, age seems to be a relevant factor associated with sedentary behavior with young adults spending more time in SB and accumulating this behavior in a higher amount of sedentary bouts compared to middle-aged adults. These findings are an indication that interventions aimed at reducing sedentary behavior could focus on different approaches for different age groups based on their respective patterns of accumulation of SB and that associations between SB patterns and cardiometabolic parameters should take age into consideration.

## Data Availability

The raw data supporting the conclusions of this article will be made available by the authors, without undue reservation.
